# Effects of SGLT2 inhibitors on patients with diabetic kidney disease: A preliminary study on the basis of podocyturia

**DOI:** 10.1111/1753-0407.13261

**Published:** 2022-02-28

**Authors:** Emre Durcan, Serbay Ozkan, Halil Ibrahim Saygi, Mevlut Tamer Dincer, Ozge Polat Korkmaz, Serdar Sahin, Cebrail Karaca, Cem Sulu, Alev Bakir, Hande Mefkure Ozkaya, Sinan Trabulus, Elif Guzel, Nurhan Seyahi, Mustafa Sait Gonen

**Affiliations:** ^1^ Division of Endocrinology and Metabolism, Department of Internal Medicine Cerrahpasa Medical School, Istanbul University‐Cerrahpasa Istanbul Turkey; ^2^ Department of Histology and Embryology Cerrahpasa Medical School, Istanbul University‐Cerrahpasa Istanbul Turkey; ^3^ Division of Nephrology, Department of Internal Medicine Cerrahpasa Medical School, Istanbul University‐Cerrahpasa Istanbul Turkey; ^4^ Department of Biostatistics and Medical Informatics Halic University Istanbul Turkey

**Keywords:** diabetic kidney disease, podocalyxin, podocyturia, SGLT2 inhibitors, synaptopodin, 糖尿病肾病, SGLT2抑制剂, 尿足细胞, synaptopodin, podocalyxin

## Abstract

**Background:**

The aim of this study was to investigate the effects of sodium glucose cotransporter 2 inhibitors (SGLT2i) on the glomerulus through the evaluation of podocyturia in patients with diabetic kidney disease (DKD).

**Methods:**

The study population was composed of 40 male patients with type 2 diabetes mellitus; 22 of them received SGLT2i (SGLT2i group), and the others who did not were the control. The DKD‐related parameters of patients were monitored before SGLT2i initiation, and then in the third and sixth month of the follow‐up period. Patients' demographic, clinical, laboratory, and follow‐up data were obtained from medical charts. Microalbuminuria was measured in 24‐h urine. The number of podocytes in the urine was determined by immunocytochemical staining of two different markers, namely podocalyxin (podx) and synaptopodin (synpo). Concentrations of urine stromal cell‐derived factor 1a and vascular endothelial growth factor cytokines were quantified with an enzyme‐linked immunosorbent assay kit.

**Results:**

At the end of the follow‐up period, decreases in glycosylated hemoglobin, glucose, systolic and diastolic blood pressure, uric acid level, and microalbuminuria, and improvement in body mass index level and weight loss were significant for the SGLT2i group. On the other hand, there was no significant difference in terms of these parameters in the control group. The excretion of synaptopodin‐positive (synpo^+^) and podocalyxin‐positive (podx^+^) cells was significantly reduced at the end of the follow‐up period for the SGLT2i group, while there was no significant change for the control.

**Conclusions:**

At the end of the follow‐up period, male patients receiving SGLT2i had better DKD‐related parameters and podocyturia levels compared to baseline and the control group. Our data support the notion that SGLT2i might have structural benefits for glomerular health.

## Graphical abstract

**Highlights**

- Diabetic kidney disease is a progressive disease with the pathology of glomerular damage and loss of podocytes.
- Sodium glucose cotransporter 2 inhibitors (SGLT2i) can be expected to decrease the amount of podocyte excretion.
- Male patients receiving SGLT2i have better diabetic kidney disease‐related parameters and podocyturia levels.

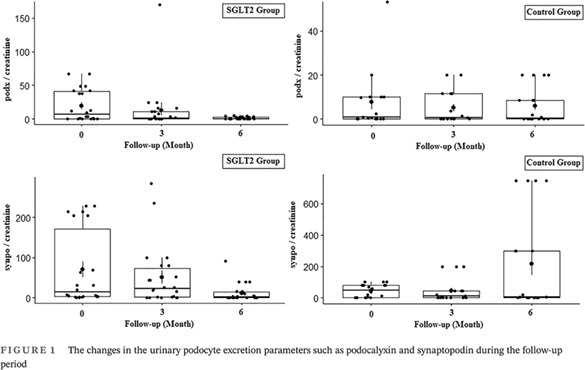

## 摘要

**背景:**

本研究旨在通过对糖尿病肾病(DKD)患者尿足细胞的评估, 探讨钠葡萄糖共转运体2抑制剂(SGLT2i)对肾小球的影响。

**方法:**

研究对象为40例男性2型糖尿病患者, 其中22例接受SGLT2i治疗(SGLT2i组), 另22例未接受SGLT2i治疗作为对照组。在SGLT2i治疗前、随访期第3个月和第6个月监测患者的DKD相关参数。从病历中获得患者的人口学、临床、实验室和随访数据。24小时中测定尿微量白蛋白。通过两种不同的标记物, 即Podocalyxin (podx)和Synaptopodin (synpo)的免疫细胞化学染色来确定尿足细胞的数量。用酶联免疫吸附分析试剂盒检测尿基质细胞衍生因子1a和血管内皮生长因子细胞因子的浓度。

**结果:**

在随访结束时, SGLT2i组糖化血红蛋白、血糖、收缩压、舒张压、尿酸水平、微量白蛋白尿均显著下降, 体重指数和体重减轻明显改善。另一方面, 对照组的这些参数没有显著差异。SGLT2i组synpo+和podx+的排泄量在随访期结束时显著减少, 而对照组无明显变化。

**结论:**

在随访期结束时, 接受SGLT2i治疗的男性患者与基线和对照组相比, 有更好的DKD相关参数和尿足细胞水平。我们的数据支持SGLT2i对肾小球健康有结构性益处的观点。

## 1 INTRODUCTION

Diabetes mellitus (DM) can lead to a broad range of microvascular complications, including nephropathy.^1^ Diabetic kidney disease (DKD) is the most common cause of chronic kidney disease (CKD), and albuminuria is considered as one of the important clinical manifestations of nephropathy.^2^, ^3^

DKD is a progressive disease with the pathology of glomerular filtration barrier (GFB) damage and loss of podocytes.^1^ Podocytes, whose cell bodies have numerous primary, secondary, and tertiary extensions which are also called foot processes, are terminally differentiated postmitotic epithelial cells.^4^ Adjacent foot processes make interdigitations, which are interconnected with slit diaphragm proteins.^4^ Slit diaphragms constitute the final barrier of the GFB.^5^ Podocytes can detach from the glomerular basement membrane (GBM) even in physiological conditions. However, with persistent insults, as in the case of hyperglycemic conditions, the detachment of podocytes could become accelerated.^6^, ^7^ Recent studies have noted that the level of podocyte excretion in urine, podocyturia, can be considered as a candidate marker for progression of glomerular pathologies.^8^, ^9^, ^10^, ^11^

Sodium glucose cotransporter (SGLT) proteins which are located in the proximal tubules are responsible for the reabsorption of glucose in the ultrafiltrate along with sodium.^12^, ^13^ Recently drugs like canagliflozin, dapagliflozin, and empagliflozin, blocking specifically glucose reabsorption of primary sodium‐coupled glucose transporters, have been developed.^14^, ^15^, ^16^ Administration of these SGLT2 inhibitors (SGLT2i) brought about reduction of blood glucose levels which is accompanied by increased excretion of glucose.^17^ Besides decreasing blood glucose, it was suggested that they could be considered as renal therapeutic agents thanks to their potential glomerular pressure‐reducing effect besides attenuating hyperglycemia‐related intraglomerular microvascular complications. As a result, administration of SGLT2i could be expected to decrease the amount of podocyte excretion. In an experimental animal model, Cassis et al showed that the SGLT2i dapagliflozin has the ability to limit podocyte damage in proteinuric non‐DKD.^8^ However, to the best of our knowledge, there is no human study examining the effects of SGLT2i on podocyturia.

In this study, we primarily aimed to investigate the effects of SGLT2i on glomerular damage through the evaluation of podocyturia in patients with DKD. In addition, we also compared the levels of urine stromal cell‐derived factor 1a (SDF‐1a) and vascular endothelial growth factor (VEGF) cytokines, which are crucial paracrine factors of intraglomerular cross talk during DM.

## 2 MATERIALS AND METHODS

### 2.1 Patients and study design

This single‐center, prospective study included 40 male patients with type 2 DM and DKD who were consecutively admitted to the Endocrinology, Metabolism and Diabetes Outpatient Clinic of Istanbul University‐Cerrahpasa, Cerrahpasa Medical School between March 2019 and September 2019. The study population was composed of two groups of patients, one of which received SGLT2i (SGLT2i group) and the other one did not (control group). The DKD‐related parameters of patients were monitored before the initiation of SGLT2i and then in the third and sixth month of the follow‐up period.

### 2.2 Study subjects and inclusion and exclusion criteria

The inclusion criteria were as follows: adult (age > 18 years) patients who had type 2 DM for at least 1 year, diagnosed with DKD, having albuminuria >30 mg/d and receiving any angiotensin‐converting enzyme inhibitors (ACEI) or angiotensin receptor blockers (ARB) for at least 6 months, with a glomerular filtration rate (GFR) > 60 mL/min and glycosylated hemoglobin (HbA1c) > 6.5. On the other hand, participants with any of the following characteristics were excluded from the study: addition of a novel drug to the current pharmacotherapy during the 6‐month follow‐up period, the presence of a clinically significant infection that causes an increase of C‐reactive protein (CRP) (more than five times the upper reference limit), presence of pyuria (more than 10 leukocytes in urine examination during the screening visit), presence of hematuria (more than five erythrocytes in a urine examination during the screening visit), presence of malignancy, and an estimated glomerular filtration rate (eGFR) less than 30 mL/min/1.73 m^2^ or acute change in kidney function, which was defined as a more than 50% change in creatinine according to the last two consecutive measurements performed during outpatient follow‐up.

In our previous study (submitted paper under review), in which we examined podocyte excretion in 396 participants (209 males, 187 females), we observed a large variability of podocalyxin‐positive (podx^+^) or synaptopodin‐positive (synpo^+^) cell excretion in females, possibly due to cell accretion from the genital tract. This large variability prevents appropriate statistical analysis. Therefore, we did not include females in this present study.

First, urine samples were collected from 25 patients in the SGLT2i group and from 23 in the control group. In total, eight participants were excluded from the study because of dropout during follow‐up. Twenty‐two patients from the SGLT2i group and 18 from the control group were included in the final analysis.

### 2.3 Data collection and definitions

Demographic, clinical, laboratory, and follow‐up data were retrieved from the medical charts of the patients and the electronic database of the hospital during the screening visit before SGLT2i initiation and in the third and sixth month of the follow‐up period. Urine samples were collected from eligible patients for the measurement of creatinine and quantification of podocytes. The following demographic and clinical data were recorded: age, duration of diabetes, body weight, body mass index (BMI), systolic and diastolic blood pressure (BP), smoking, presence of coronary artery disease (CAD), diabetic retinopathy (DR), diabetic neuropathy (DN), cerebrovascular event (CVE), and peripheral arterial disease (PAD). Recorded laboratory data consisted of measurements of glucose, HbA1c, urea, creatinine, sodium, potassium, uric acid, albumin, CRP, urine pH and sediment analysis, creatinine clearance, albuminuria, and proteinuria. Data on the use of antidiabetic drugs including oral antidiabetics and insulin were collected.

The date of urine collection was accepted as the enrollment date to the study. Diagnosis of DM was made according to the Standards of Medical Care in Diabetes‐2021 criteria.^18^ Calculations of eGFR were made according to the Chronic Kidney Disease Epidemiology Collaboration (CKD‐EPI) formula.^19^ Albuminuria and proteinuria were measured in urine collected for 24 h. Albuminuria was defined as the presence of more than 30 mg albumin/d, macroalbuminuria was defined as the presence of more than 300 mg albumin/d, and proteinuria was defined as the presence of more than 1000 mg protein/d. CAD was defined as a history of coronary artery bypass grafting or coronary angioplasty or angiographically documented CAD. DR was defined as the presence of proliferative or nonproliferative lesions according to the examination of an ophthalmologist at any time. DN was defined as electromyographic examination findings or taking medications for neuropathic pain. CVE was defined as a history of stroke resulting either from ischemic infarction of part of the brain or from intracerebral hemorrhage. PAD was defined as a history of peripheral artery bypass grafting or peripheral angioplasty or angiographically documented PAD.

### 2.4 Laboratory procedures

#### 2.4.1 Immunocytochemistry for quantification of podocytes in urine

The amount of podocytes in urine was determined as previously described by Vogelmann et al with some modifications.^9^ After a minimum of 3 h without voiding, freshly drained midstream urine samples (20‐50 mL) were collected and centrifuged at 700 g for 5 min. After discarding the supernatant, the pellet was washed twice with phosphate‐buffered saline (PBS). The final resuspension was done with 1 mL of PBS, and 100 μL of the suspension was cytospined onto a poly‐L‐lysine‐coated microscope slide with Thermo Fisher Cytospin Centrifuge 4. Air‐dried slides were fixed with a 1:1 methanol‐acetone solution for 10 min. Following incubation with 1% H_2_O_2_ solution for 15 min, the slides were subjected to heat‐induced antigen recovery in 10 mM citrate buffer solution for 15 min. The slides were treated with a blocking solution for 7 min (ScyTek Laboratories SensiTek HRP Kit, Utah) and then left overnight for incubation at 4°C with primary antibodies (1/200 and 1/100 dilution, respectively) of human anti‐sinaptopodin (Abcam, UK) and anti‐podx (Abbkine, China). After washing with PBS, the slides were incubated with biotinylated secondary antibody for 20 min and then with horseradish peroxidase (HRP)‐conjugated enzyme for 20 min (ScyTek Laboratories SensiTek HRP Kit, Utah). After treatment with 3‐amino‐9‐ethylcarbazole (AEC) chromogen, the slides were counterstained with hematoxylin. Total podx^+^ and synpo^+^ cell quantities were separately assessed and counted from all preparations under an Olympus BX61 microscope at 20X magnification by two blinded separate investigators, and two counts were averaged for each sample. We expressed the agreement between observers as Spearman's correlation coefficients based on 20 randomly selected samples. Spearman's correlation coefficient was 0.869 for podx^+^ cells and 0.971 for synpo^+^ cells. In addition, podocyte counts were standardized according to urinary creatinine excretion and expressed as the number of podocytes per mg creatinine (pod #/mg CR).^20^

#### 2.4.2 Measurement of urine SDF‐1a and VEGF

Concentrations of urine SDF‐1a and VEGF cytokines were quantified with an calorimetric enzyme‐linked immunosorbent assay (Abbkine, China) and measured with a microplate reader (Allsheng AMR‐100 Microplate Reader AS‐16050‐00, China).

#### 2.4.3 Measurement of urine creatinine

Urine creatinine was analyzed using the Roche cobas c module according to the manufacturer's kits and recommendations using enzymatic colorimetric methods (Roche cobas c 702, Roche Diagnostics GmbH, Mannheim, Germany).

### 2.5 Statistical analysis

Descriptive statistics are expressed as mean, SD, median, interquartile range, minimum, and maximum for continuous data and as counts and proportions for categorical data. Categorical data were analyzed using the chi‐square or Fisher's exact tests. The distribution normality of the continuous variables was calculated using the Shapiro‐Wilk test. We compared the two independent groups using the Student's *t* test for normally distributed variables and the Mann Whitney *U* test for nonnormally distributed variables; we compared the variables before and in the third and sixth month of the follow‐up period using the repeated measures analysis of variance (ANOVA) test for normally distributed variables and the Friedman test for nonnormally distributed variables; post hoc multiple comparison analysis was performed with significant values that had been adjusted using Bonferroni correction. Statistical analyses were performed using the IBM SPSS v. 24 for Windows software and were reported with 95% CI. Values of *P* < .05 were considered significant.

### 2.6 Ethical issues

The study was approved by the local ethics committee of Istanbul University‐Cerrahpasa, Cerrahpasa Medical Faculty (decision no.: 22722, 8 February 2019). All procedures performed in the study involving human participants were in accordance with the ethical standards of the institutional and/or national research committee and with the 1964 Helsinki Declaration and its later amendments or comparable ethical standards. Informed consent was obtained from all individual participants included in the study.

## 3 RESULTS

### 3.1 Patients' demographic, clinical, and laboratory characteristics at baseline

We examined a total of 40 patients with type 2 DM (22 in the SGLT2i and 18 in the control group) with a mean age of 61 ± 8.6 years. The demographic, clinical, and laboratory data of the patients at baseline are shown in Table 1. The groups had similar systolic and diastolic BP, and it was not well controlled in either group (>130/80 mm Hg). Also, the SGLT2i group had a high baseline eGFR of 135, meeting the definition of hyperfiltration (>125 mL/min). Clinical data along with antidiabetic drug use in each group are shown in Table S1. All of the patients had similar features for both antidiabetic pharmacotherapy and chronic diabetic complications. In addition, all of the participants had received ACEI or ARB for at least 6 months, and the diuretic drug use was similar between groups (36.4% vs 55.6%, *P* = .225). We found that an average of 87% of the maximum tolerated ACEI/ARB dose was achieved, and no dose adjustments or changes were made during the study. On the other hand, oral antidiabetic drugs lowering albuminuria such as glucagon‐like peptide 1 analogs and dipeptidyl peptidase IV inhibitors were used at maximum daily doses.

**TABLE 1:** Participants' sociodemographic characteristics and clinical and biochemical findings at baseline

Variables	SGLT2i group (n = 22)	Control group (n = 18)	*P*
	Mean ± SD		
Age, y	59.1 ± 10.5	63.4 ± 4.8	.066
Duration of diabetes, y	10 ± 5.8	11.9 ± 6.5	.299
Smoking, n (%)	6 (27.3)	2 (11.1)	.417
Body weight, kg	96.5 ± 17.4	94.2 ± 16.2	.563
BMI, kg/m^2^	32.2 ± 5.8	34 ± 6.7	.492
Systolic BP, mm Hg	135 ± 7.9	138.9 ± 5.3	.070
Diastolic BP, mm Hg	85.7 ± 6	89.7 ± 7.4	.125
HbA1c, %	8.7 ± 1	7.3 ± 0.5	**<.001**
Glucose, mg/dL	182.5 ± 65.6	136.4 ± 27.2	**.004**
Urea, mg/dL	33.3 ± 10.2	46.7 ± 19.5	.062
Creatinine, mg/dL	0.9 ± 0.2	1.2 ± 0.4	.084
Uric acid, mg/dL	4.3 ± 0.2	4.3 ± 0.2	.717
Sodium, mEq/L	139.6 ± 1.9	140.2 ± 1.7	.262
Potassium, mEq/L	4.7 ± 0.4	4.5 ± 0.4	.147
Albumin, g/dL	4.5 ± 0.3	4.6 ± 0.2	.180
CRP, mg/L	3.2 ± 2.1	4.4 ± 3.3	.427
GFR, mL/min	132.4 ± 46.3	117.3 ± 34.3	.510
Proteinuria, mg/24 h	315.7 ± 146.6	305.1 ± 165.7	.299
Microalbuminuria, mg/24 h	115.5 ± 100.6	112.9 ± 96.3	.819
Urine pH	5.8 ± 0.4	5.8 ± 0.4	.778
Urine sediment analysis, per field			
Leukocytes, median (min‐max)	0 (0‐5)	0 (0‐8)	.778
Erythrocytes, median (min‐max)	0 (0‐3)	1 (0‐3)	.396
Epithelium, median (min‐max)	0 (0‐2)	0 (0‐1)	.352
Podx/creat, median IQR (25‐75)	6.5 (0‐42)	0.8 (0‐10)	.240
Synpo/creat, median IQR (25‐75)	14 (2.5‐205)	47.2 (1.7‐82.1)	.968
SDF‐1a/creat, pg/μL, median IQR (25‐75)	0.9 (0.6‐2.3)	0.7 (0.4‐0.9)	.086
VEGF/creat, pg/μL, median IQR (25‐75)	0.8 (0‐1.8)	0.8 (0.1‐0.8)	.758

Abbreviations: BMI, body mass index; BP, blood pressure; creat, creatinine; CRP, C‐reactive protein; GFR, glomerular filtration rate; HbA1c, glycosylated hemoglobin; IQR, interquartile range; podx, podocalyxin; SDF‐1a, stromal cell‐derived factor 1a; SGLT2i, sodium glucose cotransporter 2 inhibitor; synpo, synaptopodin; VEGF, vascular endothelial growth factor.

Normalization of podocyturia with urinary creatinine is the current standard^10^, ^21^; this is why in this study podx^+^ or synpo^+^ cell counts and concentration of SDF‐1a and VEGF were reported as creatinine‐adjusted values. Podocyte counts according to the podx or synpo immune staining and concentration of urine SDF‐1a and VEGF cytokines for each group are shown in Table 1. We found no statistically significant differences in these podocyte parameters at baseline between groups (*P* > .05).

### 3.2 Changes in clinical and laboratory characteristics during follow‐up

During the follow‐up period, significant weight loss and decrease in BMI levels were detected in the SGLT2i group (*P* < .001 for all). Also, diastolic BP in the first 3 months of the follow‐up and systolic BP in the later period were significantly decreased in patients who received SGLT2i (*P* < .001 for all). However, there was no significant weight loss and changes of BP in the control group (*P* > .05). The changes in the clinical characteristics of the patients during follow‐up are summarized in Table 2.

**TABLE 2:** Changes in clinical characteristics of patients during follow‐up

	A. Baseline	B. Third month	C. Sixth month	Repeated measures ANOVA *P*	Adjusted *P* (A vs B)	Adjusted *P* (A vs C)	Adjusted *P* (B vs C)
	Mean ± SD						
SGLT2i group (n = 22)							
Body weight, kg	96.5 ± 17.4	91.4 ± 17.5	89 ± 17.7	**<.001**	**.006**	**<.001**	**.025**
BMI, kg/m^2^	32.2 ± 5.8	30.5 ± 5.8	29.7 ± 5.8	**<.001**	**.006**	**<.001**	**.025**
Systolic BP, mm Hg	135 ± 7.9	130.5 ± 7.2	125.7 ± 6.2	**<.001**	.058	**<.001**	**.004**
Diastolic BP, mm Hg	85.7 ± 6	81.4 ± 4.9	78.9 ± 3.8	**<.001**	**.016**	**<.001**	.456
Control group (n = 18)							
Body weight, kg	94.2 ± 16.2	94.2 ± 17.6	93.4 ± 17.5	.368	NA	NA	NA
BMI, kg/m^2^	34 ± 6.7	34 ± 7.1	33.7 ± 7.1	.368	NA	NA	NA
Systolic BP, mm Hg	138.9 ± 5.3	138.9 ± 5.3	138.6 ± 5.6	.368	NA	NA	NA
Diastolic BP, mm Hg	89.7 ± 7.4	89.4 ± 7.5	89.2 ± 7.7	.223	NA	NA	NA

Abbreviations: ANOVA, analysis of variance; BMI, body mass index; BP, blood pressure; NA, not applicable; SGLT2i, sodium glucose cotransporter 2 inhibitor.

While there was a significant decrease in HbA1c and glucose over time in the SGLT2i group, there was no change in the control group. In the patients who received SGLT2i, the decrease in GFR in the third month was restored in the sixth month, and GFR levels became similar to baseline GFR levels (*P* < .001 and *P* = .84, respectively). In line with these data, there was an increasing trend in creatinine levels in the third month in these patients, although it was not statistically significant (*P* = .341). While in the SGLT2i group, there was no change in the proteinuria level during follow‐up, the decrease in albuminuria was especially evident at 6 months (*P* = .096 and *P* < .001, respectively). However, these DKD‐related parameters did not change during the follow‐up period in the control group (*P* > .05 for all). The changes in the laboratory characteristics of the patients during follow‐up are summarized in Table 3.

**TABLE 3:** Changes in laboratory characteristics of patients during follow‐up

	A. Baseline	B. Third month	C. Sixth month	Repeated measures ANOVA *P*	Adjusted *P* (A vs B)	Adjusted *P* (A vs C)	Adjusted *P* (B vs C)
	Mean ± SD						
SGLT2i group (n = 22)							
HbA1c, %	8.7 ± 1	7.7 ± 0.8	7.8 ± 1.1	**<.001**	**.002**	**<.001**	1
Glucose, mg/dL	182.5 ± 65.6	131.1 ± 26.7	132.2 ± 34	**.002**	**.008**	**.01**	1
Urea, mg/dL	33.3 ± 10.2	34.8 ± 5.7	32.3 ± 9.4	.861	NA	NA	NA
Creatinine, mg/dL	0.9 ± 0.2	1 ± 0.2	0.9 ± 0.2	.341	NA	NA	NA
GFR, mL/min	132.4 ± 46.3	103.4 ± 34.3	126.6 ± 44.3	**<.001**	**<.001**	.84	**.01**
Proteinuria, mg/24 h	315.7 ± 146.6	242.5 ± 106.3	286 ± 181.2	.096	NA	NA	NA
Microalbuminuria, mg/24 h	115.5 ± 100.6	76.6 ± 73.6	65.1 ± 71.7	**<.001**	.119	**<.001**	.119
Control group (n = 18)							
HbA1c, %	7.3 ± 0.5	7.2 ± 0.6	7.1 ± 0.7	.113	NA	NA	NA
Glucose, mg/dL	136.4 ± 27.2	127.9 ± 23.4	118.8 ± 23.5	.735	NA	NA	NA
Urea, mg/dL	46.7 ± 19.5	46.4 ± 14.5	39.6 ± 8	.721	NA	NA	NA
Creatinine, mg/dL	1.2 ± 0.4	1.2 ± 0.4	1 ± 0.2	.074	NA	NA	NA
GFR, mL/min	117.3 ± 34.3	111.8 ± 30.2	115.5 ± 32.5	.139	NA	NA	NA
Proteinuria, mg/24 h	305.1 ± 165.7	304.7 ± 192.3	399.8 ± 278.9	1.0	NA	NA	NA
Microalbuminuria, mg/24 h	112.9 ± 96.3	131.3 ± 152.2	224.9 ± 199.7	.794	NA	NA	NA

Abbreviations: ANOVA, analysis of variance; GFR, glomerular filtration rate; HbA1c, glycosylated hemoglobin; NA, not applicable; SGLT2i, sodium glucose cotransporter 2 inhibitor.

### 3.3 Changes in urinary podocyte excretion parameters during follow‐up

**FIGURE 1:**
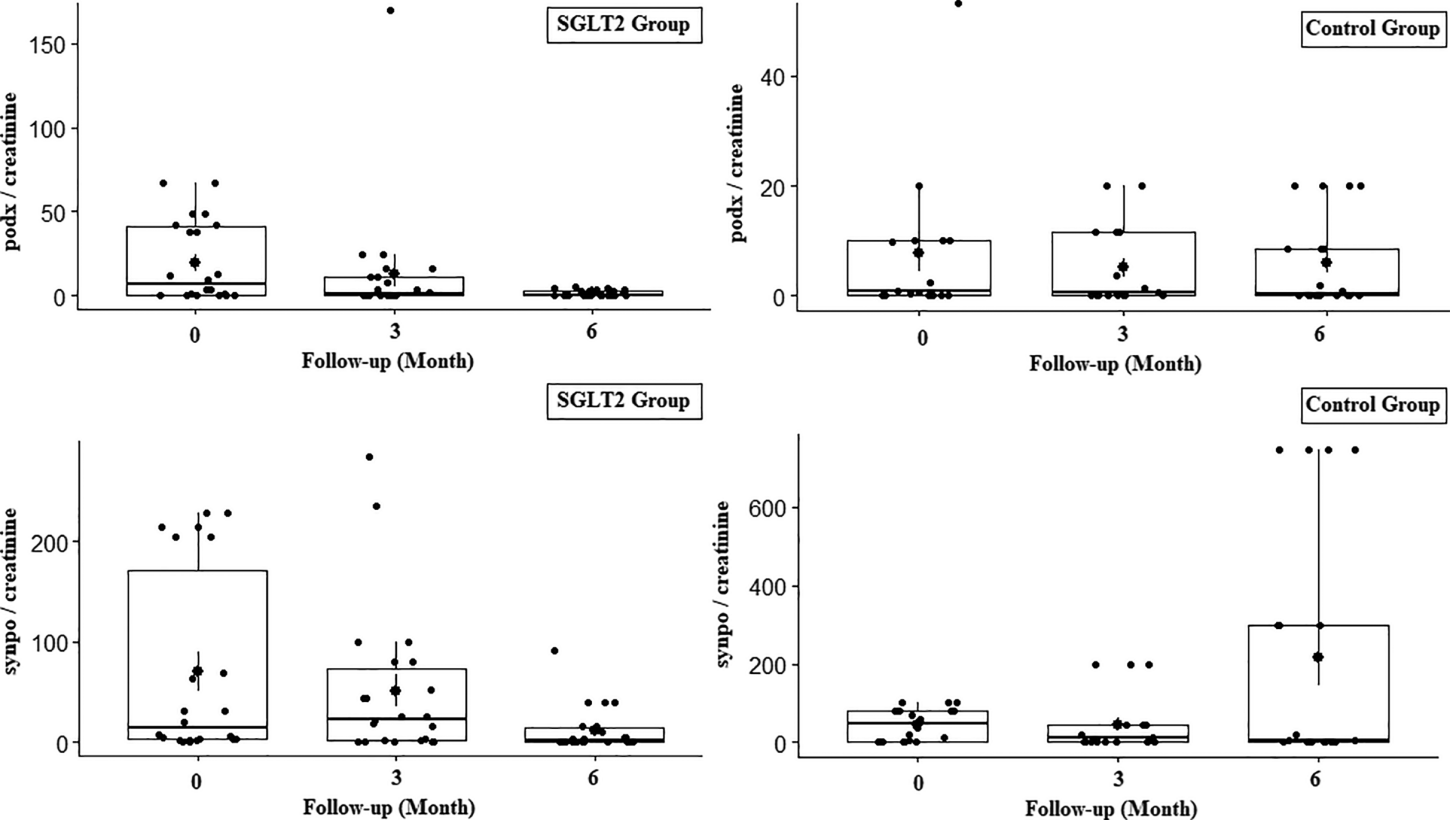
The changes in the urinary podocyte excretion parameters such as podocalyxin and synaptopodin during the follow‐up period

In the SGLT2i group, although there was no statistically significant decrease in excretion of podx^+^ cells at 3 months, a decrease was detected at 6 months (*P* = .071 and *P* = .002, respectively) (Figure 1). In addition, these patients had a significant increase in excretion of synpo^+^ cells at 3 months (*P* = .034), but the excretion of synpo^+^ cells was found to be lower than the basal level at 6 months (*P* < .001) (Figure 1). On the other hand, there was no significant change in excretion of podx^+^ and synpo^+^ cells in the control group during the follow‐up period (*P* > .05 for all). Similarly, concentrations of SDF‐1a and VEGF were at similar levels in both patient groups at any time of the follow‐up (*P* > .05 for all). The changes in urinary podocyte excretion parameters of the patients during the follow‐up are shown in Table 4.

**TABLE 4:** Changes in urinary podocyte excretion parameters of patients during follow‐up

	A. Baseline	B. Third month	C. Sixth month	Friedman test *P*	Adjusted *P* (A vs B)	Adjusted *P* (A vs C)	Adjusted *P* (B vs C)
	Median IQR (25‐75)						
SGLT2i group (n = 22)							
Podx/creat	6.5 (0‐42)	1.1 (0‐12)	0 (0‐3)	**<.001**	.071	**.002**	.775
Synpo/creat	14 (2.5‐205)	22.9 (1.2‐79.5)	1.3 (0‐15)	**<.001**	**.034**	**<.001**	**.039**
SDF‐1a/creat, pg/μL	0.9 (0.6‐2.3)	0.9 (0.4‐1.5)	1 (0.6‐1.6)	.115	NA	NA	NA
VEGF/creat, pg/μL	0.8 (0‐1.8)	0.2 (0.1.‐0.6)	1.2 (0.4‐2.7)	.135	NA	NA	NA
Control group (n = 18)							
Podx/creat	0.8 (0‐10)	0.5 (0‐11.5)	0.4 (0‐11.4)	.092	NA	NA	NA
Synpo/creat	47.2 (1.7‐82.1)	10.6 (0.5‐46)	3.8 (0‐411.5)	.576	NA	NA	NA
SDF‐1a/creat, pg/μL	0.7 (0.4‐0.9)	0.8 (0‐1)	0.9 (0.5‐1.1)	.894	NA	NA	NA
VEGF/creat, pg/μL	0.8 (0.1‐0.8)	0.9 (0.5‐2.4)	0.8 (0‐1.4)	1.0	NA	NA	NA

Abbreviations: creat, creatinine; IQR, interquartile range; NA, not applicable; podx, podocalyxin; SDF‐1a, stromal cell‐derived factor 1a; SGLT2i, sodium glucose cotransporter 2 inhibitor; synpo, synaptopodin; VEGF, vascular endothelial growth factor.

## 4 DISCUSSION

In the present study, we found that the excretion of podx^+^ and synpo^+^ cells was significantly decreased at 6 months of follow‐up in the SGLT2i group, while we did not observe any change in the control. To the best of our knowledge this is the first human study on the effects of SGLT2i by means of podocyturia. In addition, the concentration levels of SDF‐1a and VEGF were similar in all patients at any time of the follow‐up. Besides, the SGLT2i group had significantly higher weight loss, better BMI levels, and lower systolic and diastolic BP levels compared to the control and baseline. At the end of follow‐up, significant decreases in HbA1C, glucose, and albuminuria were also detected in the patients of the SGLT2i group compared to the control.

In recent years, the knowledge related to role of podocytes in the onset and progression of DKD has greatly increased. Moreover, podocyte injury and loss have been identified as key factors in the pathogenesis of DKD.^9^, ^18^, ^19^ Morphologic features of podocyte injury can be characterized as hypertrophy and effacement of foot processes, both of which bring about detachment from the GBM.^22^ Systemic insults as in the case of DKD, which is a microvascular complication, interrupt the dynamic regulation of the podocyte cytoskeleton and lead to detachment of podocytes.^6^, ^7^, ^23^, ^24^ In the present study, we used both podx and synpo to identify podocytes in the urine. Expressions of those podx and synpo proteins are markedly changed in kidneys under pathologic conditions.^25^, ^26^ Depending on the stage at which a podocyte is shed from the glomerulus, the expression levels of proteins could differ,^27^ leading to differences between podocyturia levels detected by each marker.

It is known that expression of SGLT2 proteins in patients with type 2 DM is increased, which exacerbates the existing pathologic condition.^28^ For this reason, it was thought that reducing glucose levels by inhibiting glucose reabsorption by SGLT2 proteins could be a potential therapeutic option.^29^ In this regard, clinical use of this novel strategy was shown to be effective with respect to reported data including weight loss and reductions in BP and glucose levels.^29^ In addition to all these current benefits, SGLT2i could also be effective in the treatment of DKD‐related pathologies due to the possible reduction of hyperglycemia‐related microvascular complications and reduced intraglomerular pressure mainly due to afferent arteriolar vasodilatation via tubulo‐glomerular feedback. Reduction of intraglomerular pressure is associated with reduced sheer stress in podocytes.

In the first place, the significant weight loss and improvement in BMI levels were detected in the SGLT2i group during the follow‐up period, and these changes were more prominent compared to previous reports.^30^, ^31^, ^32^, ^33^, ^34^, ^35^ This may be due to the discrepancy between clinical trial and real‐life data. On the other hand, diastolic BP in the first 3 months of the follow‐up and systolic BP in the later period were significantly decreased in patients who received SGLT2i, which is also more significant than reported in the literature.^30^, ^31^, ^32^, ^34^, ^35^, ^36^ SGLT2i cause a fluid loss of approximately 500 mL with an osmotic diuresis‐like effect, especially in the first days of treatment. This diuretic effect is likely to contribute to the BP reduction.^14^, ^17^, ^34^ Another plausible explanation is the local inhibition of the renin‐angiotensin system secondary to increased sodium distribution to the juxtaglomerular apparatus.^37^, ^38^ Compared with previous studies, the greater reduction in BP could be attributed to the greater weight loss in our study.

In the present study, the relatively short follow‐up duration might not reveal improvement in GFR as in the case of the DAPA‐CKD (Study to Evaluate the Effect of Dapagliflozin on Renal Outcomes and Cardiovascular Mortality in Patients with CKD) trial in which improvements in GFR appeared in a 12‐month long follow‐up with SGLT2i. In addition, there was no significant change in proteinuria levels during follow‐up, whereas the decrease in albuminuria was particularly evident at the end of the sixth month. In previous studies, the significant reduction in albuminuria has also been demonstrated.^36^, ^39^, ^40^, ^41^, ^42^ On the other hand, podocyturia has become the recent focus for renal function monitoring with respect to their pivotal role in the development of albuminuria.^43^ Nakamura et al identified podocytes in the urine of patients with micro‐ and macroalbuminuria, and it was also thought that depending on the severity of glomerular damage, the level of podocyturia can change.^7^ Presence of podocyturia has also been reported previously in patients with DKD.^7^, ^44^, ^45^, ^46^ Furthermore, our previous study (submitted paper under review) confirms the main findings consistent with the literature in a larger diabetic patient group.

The renoprotective effects of SGLT2i have been demonstrated in many experimental animal models, but no clinical trial data have been reported.^47^, ^48^, ^49^, ^50^ Cassis et al showed that podocytes stressed with albumin in vitro have a reduction in β1‐integrin protein expression accompanied by an increase in SGLT2 expression by remodeling of F‐actin and α‐actinin‐4 filaments. In this context, it has been shown that SGLT2i directly target podocytes and are effective by preserving the actin cytoskeleton structure.^8^ In this respect, SGLT2i were reported to ameliorate glomerular and tubular injuries induced with streptozotocin and fructose in a rat DKD model.^47^ In our study, we found that excretion of podx^+^ and synpo^+^ cells was significantly decreased at 6 months of follow‐up in the patients who received SGLT2i. However, our search for a molecular cue in urine, a possible new biomarker, did not yield a significant result. Two members of the intraglomerular cross talk factors were monitored in urine during the follow‐up period. VEGF is a critical paracrine factor between podocytes and endothelial cells, and during DKD, this cross talk was disrupted.^51^, ^52^ The other factor quantified, SDF‐1a, probably played a role in progenitor activation in the glomerulus.^53^ The concentrations of SDF‐1a and VEGF were at similar levels in these patients at any time of the follow‐up.

To the best of our knowledge, the present study with its prospective design is the first study in humans, but it has some limitations. First, the small sample size and the relatively short follow‐up period may have been inadequate to see significant changes in certain clinical and laboratory parameters. For more precise results, our findings need to be tested in larger samples of patients with DKD and longer follow‐up periods. Second, at each time point during the follow‐up period of the study, a single urine sample was taken instead of a serial urine sample. Podocytes are often shed in clusters and irregularly. Serial analysis of urine samples may be required because of this intermittent loss of podocytes in the urine.^10^ Finally, the podocyturia detection method used in this study has its own limitations.^27^ With a more precise and comprehensive detection method, podocyturia levels of patients receiving SGLT2i could be obtained, not only in males but also in females.

In conclusion, the findings of the present study indicate that male patients receiving SGLT2i had better DKD‐related parameters and podocyturia levels compared to baseline and the control group. Our data support the notion that SGLT2i might have structural benefits for glomerular health.

## CONFLICT OF INTEREST

The authors declare no conflicts of interest that could be perceived as prejudicing the impartiality of the research reported.

## AUTHOR CONTRIBUTIONS

All authors made substantial contributions to the conception and design, and/or acquisition of data, and/or analysis and interpretation of data; participated in drafting the article or revising it critically for important intellectual content; and gave final approval of the version to be submitted.

## Supporting information


**Appendix S1**: Supporting Information

## Data Availability

The data that support the findings of this study are available from the corresponding author upon reasonable request.AUTHOR: Please note that References 6 and 25, 32 and 38, and 33 and 37 were identical. We deleted the identical references and renumbered the subsequent references.
