# Supplementary material for: Effects of SGLT2 inhibitors on patients with diabetic kidney disease: A preliminary study on the basis of podocyturia
Source: J Diabetes. 2022 Feb 28;14(4):236–46. doi: 10.1111/1753-0407.13261 (PMC9060072; doi:10.1111/1753-0407.13261)
Supplement: Supplementary file 1 — Appendix S1: Supporting Information [file JDB-14-236-s001.docx]

**Table S1.** Anti-diabetic pharmacotherapy and chronic diabetic complications among study groups

| **Variables** | **SGLT2 Group**  ***(n=22)*** | **Control Group**  ***(n=18)*** | ***P*** |
| --- | --- | --- | --- |
|  | ***n (%)*** | |  |
| Anti-diabetic drugs |  |  |  |
| Metformin | 15 (68.2) | 14 (77.8) | 0.499 |
| Sulfonylureas | 7 (31.8) | 2 (11.1) | 0.119 |
| DPP-4 inhibitors | 16 (72.7) | 13 (72.2) | 0.972 |
| GLP-1 analogs | 1 (4.5) | 1 (5.6) | 0.884 |
| SGLT2 inhibitors | 22 (100) | 0 | NA |
| Pioglitazone | 1 (4.5) | 1 (5.6) | 0.884 |
| Acarbose | 1 (4.5) | 3 (16.7) | 0.204 |
| Basal insulin | 5 (22.7) | 1 (5.6) | 0.130 |
| Mixt insulin | 3 (13.6) | 5 (27.8) | 0.266 |
| Basal-bolus insulin | 8 (36.4) | 4 (22.2) | 0.332 |
| Diabetic complications |  |  |  |
| DRP | 6 (27.3) | 4 (22.2) | 0.714 |
| DNP | 6 (27.3) | 4 (22.2) | 0.714 |
| CAD | 12 (54.5) | 12 (66.7) | 0.436 |
| CVE | 2 (9.1) | 4 (22.2) | 0.247 |
| PAD | 1 (4.5) | 4 (22.2) | 0.093 |

*SGLT2*: Sodium-glucose Co-transporter, *DPP-4*: dipeptidyl peptidase-4, *GLP-1*: glucagon-like peptide, *DRP*: diabetic retinopathy, *DNP*: diabetic neuropathy, *CAD*: coronary artery disease, *CVE*: cerebrovascular event, *PAD*: peripheral artery disease, *NA*: no assessment
